# Modulation of BDNF/TrkB Signalling Pathway in Alzheimer’s Disease: Mechanistic Insights and the Role of Stem Cell Therapy

**DOI:** 10.3390/biomedicines13122931

**Published:** 2025-11-28

**Authors:** Zairin Zulaikha Harun, Auji Abdul Azhar, Yun-Jin Kim, Farah Wahida Ibrahim, Min-Hwei Ng, Jen-Kit Tan, Yogeswaran Lokanathan

**Affiliations:** 1 Department of Tissue Engineering and Regenerative Medicine, Faculty of Medicine, Universiti Kebangsaan Malaysia, Cheras, Kuala Lumpur 56000, Malaysia; 2 Center for Toxicology & Health Risk Studies, Faculty of Health Sciences, Universiti Kebangsaan Malaysia, Kuala Lumpur 50300, Malaysia; 3 School of Traditional Chinese Medicine, Xiamen University Malaysia, Sepang 43900, Selangor, Malaysia; 4 Advance Bioactive Materials-Cells UKM Research Group, Universiti Kebangsaan Malaysia, Bangi 43600, Selangor, Malaysia; 5 Department of Biochemistry, Faculty of Medicine, Universiti Kebangsaan Malaysia, Cheras, Kuala Lumpur 56000, Malaysia

**Keywords:** stem cell therapy, Alzheimer’s disease, BDNF, TrkB, signalling pathway, synaptic plasticity, cognitive functions

## Abstract

Alzheimer’s disease (AD) is a progressive neurodegenerative disease, characterized by the accumulation of amyloid beta (aβ) plaques and neurofibrillary tangles, along with progressive deterioration of cognitive function. AD is the most common form of dementia and affects over 55 million people worldwide. Current treatments for AD are symptomatic-based rather than curative, which calls for the development of new therapeutic strategies. Stem cell therapy has shown promising results for neurodegenerative diseases, including AD. Brain-derived neurotrophic factor (BDNF) and its receptor, tropomyosin receptor kinase B (TrkB), and their downstream signalling cascades play crucial role in modulating neuronal survival, development and synaptic plasticity, which are vital for cognitive functioning, and this pathway is dysregulated in AD. While the BDNF/TrkB signalling pathway dysregulation and stem cell therapy are each widely studied in AD, the interplay between those two remains underexplored. This review focuses on the mechanistic insights of the BDNF/TrkB signalling pathway in normal physiological condition and AD, along with the effects of stem cell therapy on the pathway and its downstream cascades. The findings highlight the therapeutic outcomes in increasing BDNF/TrkB levels and functions, restoring synaptic plasticity, modulating downstream substrates activities and improving cognitive functions. In addition, challenges, limitations and future directions of stem cell therapy are discussed, underscoring the therapeutic benefits of this therapy for AD by modulating the BDNF/TrkB signalling pathway.

## 1. Introduction

Neurodegenerative diseases (NDs) are progressive disorders that affect the human brain, particularly in the elderly. According to the Global Burden of Disease Study 2021, the number of people affected by neurological conditions in 2021 was estimated at around 3.4 billion worldwide, accounting for 43.1% of the world population [1]. The incidence of NDs is expected to increase in parallel with the ageing population, as most of the NDs are strongly correlated with ageing [2]. NDs are defined by the gradual degeneration and loss of neurons in the central nervous system (CNS) or peripheral nervous system (PNS). The disruption to the structure and function of the neurons eventually leads to impairment in memory, cognition, behaviour, sensory and/or motoric functions [3]. Alzheimer’s disease (AD) is a prominent example of NDs. Among all NDs, AD is the most common, accounting for more than 55 million cases worldwide according to the World Health Organization (2023).

Proteinopathy, or misfolding and abnormal aggregation of proteins, is a way to classify NDs and is one of the key hallmarks in the progression of diseases such as AD [4]. Aggregation and functional loss of specific proteins are common features in most NDs. Misfolded proteins tend to accumulate together and form large and insoluble aggregates. The causes of protein aggregation may be due to mutations, mislocalization, posttranslational modifications or other factors [5]. In AD, aggregation of amyloid beta (aβ) protein, which is caused by abnormal cleavage of amyloid precursor protein (APP), and deposition of neurofibrillary tangles (NFT), which is caused by hyperphosphorylation of tau protein, can be observed [6]. In addition to proteinopathy, NDs like AD also exhibit other common hallmarks such as synaptic and neuronal network dysfunction, neuroinflammation, DNA and RNA defects, abnormal proteostasis, cytoskeletal abnormalities, altered energy homeostasis, neuronal cell death and cognitive impairments [3].

Current therapeutic strategies for AD mainly focus on alleviating the symptoms experienced by the patients. The current treatments for AD are primarily targeting neurotransmissions by mimicking neurotransmitters or by inhibiting/promoting neurotransmitters [7]. The approved drugs by the Food and Drug Administration (FDA) for AD are acetylcholinesterase inhibitors (donepezil, rivastigmine and galantamine), N-methyl-D-aspartate (NMDA) receptor antagonist (memantine) and anti-aβ immunotherapies (aducanumab and lecanemab) [8]. The blood–brain barrier (BBB), a protective barrier of the brain, remains as a major challenge in developing effective drug delivery to the brain, along with the complexity of the disease mechanisms, as administration of the drugs are mostly at the late stages of AD [9]. Promoting neuronal health and preventing neurodegenerative changes at the molecular level serve as the goal in exploring and developing treatments for NDs [10].

Brain-derived neurotrophic factor (BDNF) is a member of the neurotrophin family and plays essential roles in maintaining neuronal health and synaptic function in the CNS. Synthesis of BDNF begins in the cell bodies of neurons and glia and is then transported and released at the neuron terminals. BDNF has a high affinity to bind to the tropomyosin receptor kinase B (TrkB) receptor. The BDNF receptor, TrkB, is a member of the receptor tyrosine kinase (RTK) superfamily. TrkB is an essential structure and widely expressed by all neurons. TrkB is located within the Golgi complex and endocytic vesicles of the neurons and is then translocated to the plasma membrane after neuronal activation [11]. Upon binding of BDNF to TrkB, it will induce receptor homodimerization and activation, which will then further trigger signalling pathways, such as PI3K/Akt, MAPK/ERK and PLC-γ pathways. These pathways are crucial for neuronal developmental, survival and plasticity. Dysregulation of the BDNF/TrkB signalling pathway in AD contributes to cognitive deficits such as learning impairment and memory loss [12,13].

Over the past decades, stem cell studies have made a significant impact in developing regenerative medicine, on top of unravelling knowledge for the complex mechanisms in human development [14]. Regenerative medicine focuses on regenerating, repairing, improving or replacing cells, organs or tissues impaired and/or providing trophic support in abnormalities such as ageing and diseases. The use of stem cells from different sources in regenerative medicine is focused on cellular therapy to replace the damaged cells [15]. Stem cells, with their ability to self-renew and differentiate into various cell types, have seen a substantial growth in studies over the years, focusing on multiple diseases including AD [16,17]. Neural stem cells (NSC), mesenchymal stem cells (MSC), induced pluripotent stem cells (iPSC) and embryonic stem cells (ESC) are examples of stem cells that are studied for the treatment of AD. In clinical trials, the primary focus is on the use of adult stem cells such as NSC and MSC, together with iPSCs and their derivatives [18]. The therapeutic potentials of stem cell therapy in AD are mainly attributed to the improvement of cognitive functions, regulation of paracrine and neurotrophic signalling, modulation of neuroinflammation, enhancement of autophagy and promotion of endogenous repair mechanisms to the compromised nervous system [19].

While the findings of stem cell therapy for AD are promising, the influence of stem cells specifically on the BDNF/TrkB signalling pathway and its downstream cascades has not been thoroughly analyzed. This paper presents a review of stem cell therapies for AD focusing on its action on the BDNF/TrkB signalling pathway and its downstream cascades, particularly the PI3K/Akt, MAPK/ERK and PLC-γ signalling pathways. We will provide an overview of the BDNF/TrkB signalling pathway and its dysregulation in AD, followed by the key preclinical and clinical findings of stem cell therapy and the evidence of its mechanistic action via the BDNF/TrkB signalling pathway. Research gaps and future prospects of stem cell therapy for AD will be discussed toward the end.

The articles included for the effects of stem cell therapy on the BDNF/TrkB signalling pathway were selected based on their specific investigations of stem cell transplantation on the BDNF/TrkB axis and its downstream cascades in in vivo and in vitro models of AD. Studies on other disease models and those that did not directly address the therapeutic effects of the engrafted stem cells on the BDNF/TrkB signalling and its downstream cascades were excluded.

## 2. Overview of BDNF/TrkB Signalling Pathway

BDNF is a member of the neurotrophin family and was first discovered and purified by [20]. BDNF is widely expressed in various parts of the brain such as the hippocampus, amygdala, cerebral cortex and cerebellum, with the highest expression detected in the hippocampus followed by the cerebral cortex [21]. Other than BDNF, other neurotrophins are nerve growth factor (NGF), neurotrophin-3 (NT-3) and neurotrophin-4 (NT-4). Among all neurotrophins, BDNF is the most abundant and thoroughly studied [22]. The three-dimensional structure of BDNF consists of two pairs of antiparallel β-strands and cysteine residues in a cystine knot motif, same as NGF, NT-3 and NT-4. About 50% of BDNF’s amino acid identity is also shared with other neurotrophins. Each neurotrophin binds to its specific receptor(s), also known as tropomyosin-related kinase (Trk) receptors. For BDNF, it has high affinity for TrkB and lower affinity for p75 neurotrophin receptor (p75NTR) which all neurotrophins also bind to [23,24].

BDNF is firstly synthesized in the form of its precursor known as preproBDNF (32–35 kDa), which has three sequences: signal sequence, prodomain and mature domain in the endoplasmic reticulum. The signal sequence is rapidly cleaved after translocation to the Golgi apparatus, which forms proBDNF (28–42 kDa). proBDNF is then cleaved to form active isoforms, which are BDNF pro-peptide and mature BDNF (mBDNF) (13 kDa). The cleavage of BDNF occurs intracellular and extracellularly. Both proBDNF and mBDNF are released following cell membrane depolarization in neuronal cells and bind to TrkB and p75NTR, respectively [25,26].

TrkA, TrkB and TrkC make up the Trk family, which belongs to the receptor tyrosine kinase (RTK) superfamily. TrkA was the first to be discovered as the receptor for NGF, followed by TrkB as a receptor for BDNF, NT-3 and NT-4, and also TrkC as the receptor for NT-3 [27,28,29]. TrkB is a single-pass transmembrane protein which has extracellular domain (ECD) for binding of neurotrophins, a transmembrane domain (TMD) for transmission of signal across the cell membrane and an intracellular tyrosine kinase domain (TKD) for activation of intracellular signalling pathways. The structures of each individual domain have been described, but the full-length TrkB structure is yet to be resolved [11]. The NTRK2 gene located on the chromosome 9q22.1 is responsible for encoding full-length TrkB (TrkB.FL) and three truncated forms (TrkB.T1, TrkB.t2 and TrkB-T-Shc). The truncated forms of TrkB lack TKD [30,31].

In the absence of neurotrophin signalling, Trk receptors are mainly retained within intracellular vesicles. Their translocation to the plasma membrane is triggered by stimuli such as calcium influx, elevated cyclic adenosine monophosphate (cAMP), or neuronal depolarization, which promote the exocytosis of these vesicles. Specifically, for the TrkB receptor, neuronal activity drives calcium entry through AMPA and NMDA receptors. This calcium signal stimulates adenylyl cyclase to increase intracellular cAMP, which in turn activates both the PKA and PI3K pathways. These coordinated signalling events facilitate the microtubule-dependent mobilization of intracellular TrkB receptors to the cell surface. Beyond this vesicular trafficking, the downstream signalling capacity of Trk receptors is further modulated by several mechanisms: alternative splicing of Ntrk2 that generates truncated isoforms, Ca^2+^ and cAMP regulated vesicular insertion, and interactions with the p75NTR [32,33]. p75NTR regulates the activity of Trk receptors by binding to the non-preferred neurotrophin. For TrkB, it has been found that co-expression with p75NTR increases the specificity of TrkB activation through binding with BDNF compared to binding with NT-3 and NT-4 [34].

The ECD of TrkB consists of cysteine clusters, leucine-rich repeats and immunoglobulin-like domains (Ig). The binding of BDNF to TrkB occurs at the ECD, specifically at the second immunoglobulin-like domain (Ig2), which is the major ligand binding interface for TrkB [33]. Following BDNF binding, the BDNF-TrkB complex is internalized from the plasma membrane via two primary pathways: clathrin-mediated endocytosis and macropinocytosis mediated by cell surface ruffles. After internalization, the complex is subsequently localized to endosomal compartments. The binding of BDNF to TrkB initiates the dimerization and autophosphorylation of the tyrosine regions [33,35,36,37].

**Figure 1:**
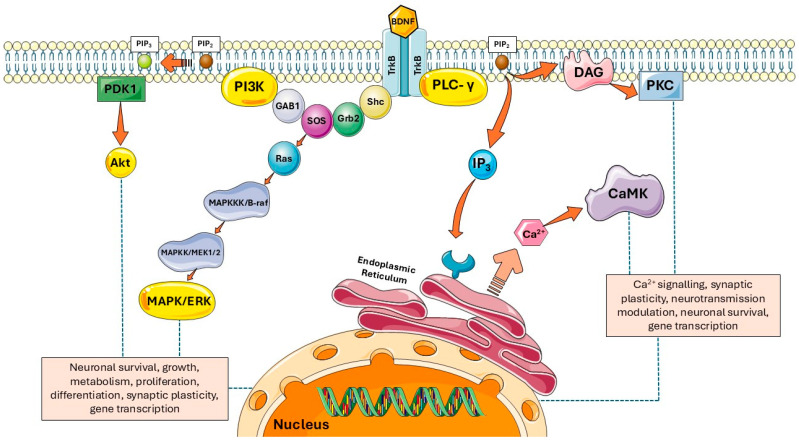
Overview of BDNF and TrkB signalling pathways. Upon binding of BDNF to TrkB, it will induce receptor homodimerization and activation which will then further trigger signalling pathways, such as PI3K/Akt, MAPK/ERK and PLC-γ pathways. These pathways are crucial for neuronal survival and developmental and synaptic plasticity. Image(s) adapted from Servier Medical Art (https://smart.servier.com/), licensed under CC BY 4.0 (https://creativecommons.org/licenses/by/4.0/).

The autophosphorylation of TrkB forms a docking site for the protein (Src-homology 2) domain containing adaptor protein (Shc) and phospholipase-C (PLC). Docking of Shc to the activated receptor is followed by interaction to other adaptor proteins such as (Grb2). The Shc/Grb2 complex then recruits Son of Sevenless (SOS), a guanine nucleotide exchange factor of Ras. Another protein that is recruited and interacts with the complex is known as GAB1 or the Grb2-associated-binding protein 1. The recruitment of these adaptor proteins is important in the activation of the downstream pathways such as the PI3K/Akt and MAPK/ERK pathways as shown in Figure 1 [38,39,40,41,42].

Postendocytic movement of TrkB is mainly through either a degradative pathway by lysosomes or a recycling pathway to the plasma membrane. Ubiquitination of TrkB is associated with a reduction in receptor number at the cell surface and ligand response. Recycling of TrkB receptors is dependent on the types of TrkB. TrkB.FL requires involvement of hepatocyte growth factor-regulated tyrosine kinase substrate (Hrs), while the truncated forms of TrkB, such as TrkB.T1, mainly go through the default recycling mechanism with involvement of the Rab family GTPase [43,44].

### 2.1. PI3K/Akt Signalling Pathway

The PI3K/Akt signalling pathway is important in growth, differentiation, survival and apoptosis of neurons and regulation of metabolism. Phosphoinositide 3-kinase (PI3K) is a member of the intracellular lipid kinase family and is composed of p55 and p85 regulatory subunits and p110 catalytic subunit [45]. PI3K is divided into three categories according to the different subunits and substrates with Class 1 of the PI3K, which consists of p85 and p110 subunits, being the most important in signalling among others [46]. PI3K is recruited to the series of receptor-associated adaptor proteins (Shc-Grb2-SOS-GAB1) attached to the activated TrkB receptor. Upon signal transduction from upstream, the p110 catalytic subunit of PI3K catalyzes the plasma membrane lipid, phosphatidylinositol-4,5-bisphosphate (PIP_2_), to generate phosphatidylinositol-3,4,5-triphosphate (PIP_3_). PIP_3_ enables the recruitment of phosphoinositide-dependent kinase (PDK1) to the membrane which will then phosphorylate Akt. Phosphorylation of Akt enhances its kinase activity, resulting in phosphorylation of downstream signalling molecules [47,48,49]. Activated Akt and its downstream substrates play crucial roles in regulating various cellular functions such as growth, proliferation, survival and apoptosis of cells, gene transcription, protein synthesis, angiogenesis and metabolism [50]. One of the substrates targeted by Akt is glycogen synthase kinase 3β (GSK-3β), where its activity is inhibited by Akt through phosphorylation at the Ser21 and Ser9 residues [51]. Phosphorylated Akt has also been shown to regulate gamma-aminobutyric acid (GABA) receptor type A, which is vital for fast inhibitory synaptic transmission [52]. Partial activation of Akt has also been shown to result in memory formation and synaptic plasticity via induction of long-term potentiation (LTP) [53]. One of the Akt downstream substrates, mammalian target of rapamycin (mTOR), is the key mediator in the regulation of autophagy and neuroprotective effects of BDNF through autophagy is associated with PI3K/Akt/mTOR signal transduction [54].

### 2.2. MAPK/ERK Signalling Pathway

The MAPK/ERK signalling pathway is important in regulating the gene transcription important for neuronal growth, differentiation and survival, and synaptic plasticity for memory formation and learning. The MAPK/ERK pathway is the primary pathway instigated by the MAPK signalling. The mitogen-activated protein kinase (MAPK) is part of the serine/threonine protein kinases family that plays a crucial role in cellular signalling processes. The initiation of this pathway begins with activation of a G protein known as Ras by the adaptor protein SOS, attached together at the activated TrkB with the other adaptor proteins such as Grb2. Ras is activated through the exchange of guanosine diphosphate (GDP) to guanosine triphosphate (GTP) promoted by SOS [55]. Activated Ras will then further trigger the kinase cascade involving other molecules, starting with activation of B-raf (also known as MAPKKK). B-raf then phosphorylates MEK1/2 (also known as MAPKK), a dual-specificity kinase that is a known activator of ERK. ERK stands for extracellular signal-regulated kinase and is also known as MAPK [56,57]. ERK can translocate to the nucleus upon its activation to regulate cell cycles’ important factors for transcription and translation such as E26 transformation-specific (ETS)-like transcription factor 1 (Elk-1), cAMP response element-binding protein (CREB), ribosomal protein S6, eukaryotic initiation factor 4E (eIF4E) and eIF4E-binding protein 1 (4E-BP1). These factors play important roles in neuronal activity, synaptic plasticity, gene transcription and protein translation [58,59,60].

### 2.3. PLC-γ Signalling Pathway

The phospholipase C-γ (PLC-γ) signalling pathway is important in regulating calcium signalling, neuronal survival and gene transcription, and promoting synaptic plasticity. PLC-γ is part of the PLC enzyme family and is recruited and phosphorylated by activated TrkB. Tyrosine residue 785 (Tyr785) on activated TrkB acts as the docking site for PLC-γ via its SH2 domain [35]. Activated PLC-γ hydrolyzes PIP_2_ at the inner leaflet of the plasma membrane to generate second messengers which are inositol 1,4,5-triphosphate (IP_3_) and diacylglycerol (DAG). IP_3_ travels to the endoplasmic reticulum (ER) where it binds with the IP_3_ receptor on ER and facilitates the release of Ca^2+^ ions into the cytoplasm [61,62]. The released Ca^2+^ ions can further bind to calmodulin, activating calcium/calmodulin-dependent protein kinases (CaMK). The CaMKs play important physiological roles, including modulation of LTP by increasing the synaptic strength, activation of CREB for transcription, neuronal memory and many more [63]. The other second messenger, DAG, remains in the plasma membrane and activates protein kinase C (PKC). PKC can modulate synaptic plasticity by regulating α-amino-3-hydroxy-5-methyl-4-isoxazolepropionic acid (AMPA) and NMDA receptors at postsynaptic levels, regulate cytoskeleton dynamics by controlling the phosphorylation of tau protein and GSK-3β, and promote actin depolarization through phosphorylation of myristoylated alanine-rich C-kinase substrate (MARCKS) and axonal membrane protein GAP-43 [64,65].

## 3. Dysregulation of BDNF/TrkB Signalling Pathway in AD

### 3.1. Pathophysiology of AD

AD is the most common form of dementia, and the main hypothesized pathophysiology of AD is the accumulation of aβ plaques and NFT [66,67]. The aβ plaques are formed due to the abnormal cleavage of amyloid precursor protein (APP) by the β- and γ-secretases instead of α- and γ-secretases in normal condition [68]. The cleavage forms aβ oligomers that tend to aggregate together, forming senile plaques. The aβ plaques formed can trigger immune activation that leads to neuroinflammation and damage to the tissue and also cause impairment of synaptic transmission [69,70]. The aβ plaques also trigger hyperphosphorylation of tau proteins [71]. Tau proteins are important in maintaining the cytoskeletal integrity of neurons as they hold and stabilize the microtubules. Hyperphosphorylation of the tau proteins causes them to be detached from the microtubules structure, clump together and form aggregates inside the neurons, also known as NFT. The NFT causes damage to the neurons and eventually leads to degeneration of neurons [72,73]. Other hallmarks of AD include cognitive deficits, neuronal loss, dysfunction of mitochondrial and autophagy, neuroinflammation and cholinergic insufficiency [74,75].

### 3.2. Reduction in BDNF and TrkB Levels in AD

Studies have shown reduced expressions of BDNF and TrkB in AD [76,77,78]. The reduction in pro-BDNF and mature BDNF levels occurs from the preclinical stage of the disease [79]. The cell death mechanisms of ferroptosis and pyroptosis are also linked to alteration of BDNF level in neurological conditions and neurodegeneration including AD [80]. Epigenetic factors such as DNA methylation, histone modification and miRNA regulation also contribute to altering BDNF levels [81]. The BDNF promoter exhibits hypermethylation, which correlates negatively with cognitive test scores, indicating transcriptional silencing [82]. Besides that, downregulation of miR-132 in AD is also associated with BDNF, as BDNF can induce the expression of miR-132. Another miRNA involved in the epigenetic mechanism of BDNF in AD is miR-206, a direct post-transcriptional repressor of BDNF. In AD, miR-206 is upregulated, further driving BDNF deficiency [83]. Impaired BDNF has also been found to cause memory impairment in cells and animal models [84]. Reduced levels of BDNF and TrkB will also cause disruption to the downstream cascades activated by the BDNF/TrkB pathway.

### 3.3. BNDF/TrkB Dysregulation Effects on Downstream Cascades—PI3K/Akt Signalling Pathway

One of the downstream cascades affected is the PI3K/Akt signalling pathway. Reduced Akt in AD can lead to lifted inhibition of GSK-3β. GSK-3β is then able to promote the phosphorylation and aggregation of tau which contributes to the progression of AD [85,86]. GSK-3β activity can be induced by aβ plaques which will further impair the activation of Akt and also contribute to the accumulation of aβ plaques [87,88]. GSK-3β also facilitates the activation of apoptotic signalling cascades [89]. Dysregulation of PI3K/Akt signalling in AD has also been shown to contribute to inflammation through modulation of cytokines, increase in oxidative stress through mitochondrial dysfunction, and dysregulation of cholinergic neurotransmission through modulation of acetylcholine (Ach) activity [90,91,92]. Disruption of PI3K/Akt also interferes with autophagy through regulation of mTOR, where altered mTOR is linked to GSK-3β and autophagy functions, and facilitates tau pathology in AD [93].

### 3.4. BNDF/TrkB Dysregulation Effects on Downstream Cascades—MAPK/ERK Signalling Pathway

Dysregulation of the BDNF/TrkB pathway also disrupts MAPK/ERK signalling. ERK has been found to negatively modulate β-secretase expression. However, under conditions like oxidative stress, the neuroprotective effect of ERK fails, which leads to overproduction of aβ [94]. It has also been proven that the aβ oligomers are able to reduce ERK and CREB activities which can contribute to cognitive decline in AD, as those components are important in learning and memory formation [95]. Deprivation of ERK activation has been shown to be associated with negative effects on synaptic plasticity directly caused by aβ plaques [96]. The MAPK/ERK pathway is also correlated with hyperphosphorylation of tau, where increased ERK activation can be observed related to progression of tau tangles in AD [97,98]. ERK alteration and overactivation is linked to memory deficits where inhibition of ERK results in reversal of memory impairment seen in AD models [99].

### 3.5. BDNF/TrkB Dysregulation Effects on Downstream Cascades—PLC-γ Signalling Pathway

Dysregulation in the PLC-γ signalling pathway is linked with tau proteins, where tau and arachidonic acid have been found to activate PLC-γ, and the interaction of tau and the SH3 domain of PLC-γ suggests the involvement of tau in PLC-γ signal transduction [100,101]. PLC-γ level is also significantly lower in AD cortical tissue compared with controls [102]. The level and activity of PKC, one of the substrates activated through this axis, have been found to be significantly decreased in AD [103,104]. The accumulation of aβ plaques in AD has been observed to downregulate PKC [105]. As the PLC-γ signalling pathway is involved in the regulation of Ca^2+^ signalling in ER through activity of IP_3_, dysregulation of this axis can cause alteration and disruption to the Ca^2+^ signalling, which further contribute to the AD pathology. Interference with APP proteolytic activity, disturbance of unfolded protein receptors, involvement in apoptotic cascades, alteration of local circuit activity and disruption of neuronal activity and synaptic plasticity caused by altered Ca^2+^ regulation contribute to AD pathology [106].

### 3.6. BDNF/TrkB Dysregulation Effects on Neuroinflammation and Neuronal Apoptosis

The downregulation of BDNF/TrkB signalling in AD is also associated with the promotion of neuroinflammation and neuronal apoptosis. BDNF has been shown to exert neuroprotective effects through suppression of microglia and astrocytes activation, downregulation of pro-inflammatory cytokines and upregulation of anti-inflammatory cytokines [107,108]. The mechanism of the BDNF/TrkB axis in neuroinflammation is also associated with the activation of the JAK-STAT pathway. The JAK-STAT pathway also increases the expression and activity of δ-secretase in turn through the upregulation of its transcription factor, C/EBPβ, which leads to the cleavage of APP and tau that contributes to the formation of aβ plaques and NFT [109]. Reduced TrkB level also gives rise to BDNF-mediated activation of p75NTR which can mediate neuronal apoptosis [110,111].

## 4. Effects of Stem Cell Therapy on BDNF/TrkB Signalling Pathway in AD

### 4.1. Upregulation of BDNF/TrkB Levels and Enhanced Cognitive Functions

Enhanced BDNF level in AD is closely linked to improved cognitive functions, particularly learning and memory formation through behavioural assessments such as novel object recognition test (NORT), open field test (OFT), Y-Maze test, Morris water maze test (MWM), elevated plus maze test (EPM), tail suspension test (TST) and many more. The underlying mechanisms involved were further clarified through biochemical analyses of genes, proteins or substrates of interest. Multiple studies have observed the enhancement of BDNF level post transplantation of stem cells in AD models, which contributes to cognitive function repair and trophic support effects mediated by BDNF [112,113,114,115,116]. These pieces of evidence clearly show that upregulation of the BDNF/TrkB pathway improves cognitive functions in AD models.

A study was conducted by Liu et al. [117], where lateral ventricle administration of exosomes derived from bone marrow MSCs (BMSC-exos) into the STZ-injected AD mice model increased the BDNF level and improved behavioural performance. It was also worth noting that caudal vein injection or intravenous injection (IV) of BMSC-exos was also performed in that study; however, no significant differences in BDNF level and behavioural performance compared with the lateral ventricle injection or intracerebroventicular injection (ICV) of the BMSC-exos could be observed, which highlights the importance of the administration route of the stem cells for in vivo models. BDNF/TrkB expressions were found to be significantly increased post transplantation of NSCs into APP/PSI transgenic mice via stereotactic delivery in a study by Zhang et al. [118]. The cognitive deficit was also significantly restored in the NSC-treated group in the study. It could be observed that improvement in cognitive ability is linked to the increase in BDNF/TrkB, as this pathway plays a crucial role in memory acquisition and consolidation by promoting synaptic plasticity and neuronal growth and survival.

In a study by Blurton-Jones et al. [119], NSCs were transplanted via stereotactic delivery into the hippocampus of the transgenic model of AD (3xTg-AD). It has been found that the NSC-treated group performed better in MWM and NORT compared to the vehicle group, which indicates improvement in memory formation. Interestingly, the study also found that there is no significant improvement in ameliorating the aβ and tau pathology, indicating that cognitive improvement was not due to alteration of aβ plaques and NFT. Instead, it was noted that there were significant differences in BDNF levels and synaptic density of the NSC-treated mice where both levels were elevated. It was further confirmed that cognitive effects of the NSC treatment were associated heavily with NSC-derived BDNF, as the knockdown of BDNF in NSCs before transplantation did not result in cognitive rescue in the AD model. This further emphasizes the importance of BDNF regulation in memory enhancement for AD.

### 4.2. Enhanced Downstream Cascades of BDNF/TrkB Signalling Pathway

Transplantation of human NSCs via lateral ventricle into NSE/APPsw transgenic mice reported a significant increase in BDNF, TrkA/B and Akt levels [120]. A decrease in tau phosphorylation observed in that study is linked with Trk-dependent Akt/GSK-3β signalling, where an increase in Akt facilitates the inhibition of GSK-3β, which promotes tau phosphorylation. Downregulation of aβ production due to reduced BACE1 expressions mediated via Akt/GSK-3β signalling, together with reduced neuroinflammation, enhanced synaptic plasticity and anti-apoptotic functions via trophic support, also contribute to the improvement of spatial memory. However, it was also mentioned that the transplantation ameliorated the impaired spatial memory but did not prevent long-term progressive cognitive impairment in the transgenic model.

A study by Gaber et al. [121] also focused on the Akt/GSK-3β pathway, where it showed that IV of bone marrow-derived MSCs (BMSCs) into Aβ25-35-induced AD rat dams during pregnancy was able to reverse the downregulation of BDNF and upregulation of GSK-3β levels at postnatal age, which contribute to the mitigation of AD. An in vitro study of MSC’s effects on aβ-treated neural cells showed positive effects through modulation of mTOR, AMPK, GSK-3β and Wnt/β-catenin pathways which are related to AD pathology, suggesting therapeutic potential of MSC therapy on AD [122]. Downregulation of GSK-3β activity which is linked to PI3K/Akt and Wnt3a-βcatenin signalling, along with enhanced neurogenesis and cognitive functions, are observed through transplantation of BMSCs together with CX3C motif ligand 1 (CX3CL1), a neuron secreted chemokine, and Wnt3, principal regulator of hippocampal neurogenesis into APP/PS1 transgenic mouse via lateral ventricle, as reported by Li et al. [123].

Xiong et al. [124] reported that human dental pulp stem cells (hDPSCs) exert neuroprotective effects focusing on oxidative stress likely attributable to the Akt/GSK-3β-mediated Nrf2 activation in both in vitro and in vivo models of AD. Suppression of GSK-3β was also able to ameliorate neuroinflammation through conversion of microglia and downregulation of pro-inflammatory mediators’ secretion [125]. These studies highlight the therapeutic potential of the stem cell therapy in AD and its role in the modulation of the PI3K/Akt pathway, particularly in the suppression of GSK-3β expression.

Modulation of PI3K/Akt/mTOR signalling by MSC-derived exosomes in AD has been studied recently by [126]. One of the mechanisms of AD amelioration by the exosomes in the study is through regulation of autophagy. The hyperactivation of PI3K/Akt/mTOR resulted in decreased autophagy in the aluminum-induced AD rats’ brains, and the findings showed the restoration of this axis in MSC-derived exosomes together with the mTOR inhibitor group, as can be seen through increased autophagy activity along with decreased APP cleavage, increased proteolytic degradation of aβ and improved memory performance. Yu et al. [127] reported that transplantation of BMSCs was able to enhance the Selective Alzheimer’s disease indicator-1 (Seladin-1), the neuroprotective effector and specific AD marker, and nestin, the cell proliferation marker, in the aluminum-induced AD rat model. The increase in these two components was mentioned to be possibly linked to the activation of PI3K/Akt and MAPK/ERK signalling pathways, as the finding showed that transplantation of BMSCs managed to increase Akt and ERK expressions.

Neuroprotection effects of BMSCs against aβ-induced apoptosis through enhanced MAPK/ERK signalling in hippocampal neurons were observed through increased levels of ERK and CREB in a study by Lee at al. [128]. In the same study, the effects of BMSC transplantation were also investigated in the aβ-induced mice model. Transplantation of BMSCs was able to mitigate AD through reduced oxidative stress and neuroinflammation and improved cognitive functions. Another study by Banik et al. [129] reported that transplantation of human umbilical-cord-blood-derived lineage negative stem cells was able to exert the neuroprotective mechanism through upregulation of BDNF and CREB along with improved spatial memory function observed in the aβ-induced mouse model.

**Figure 2:**
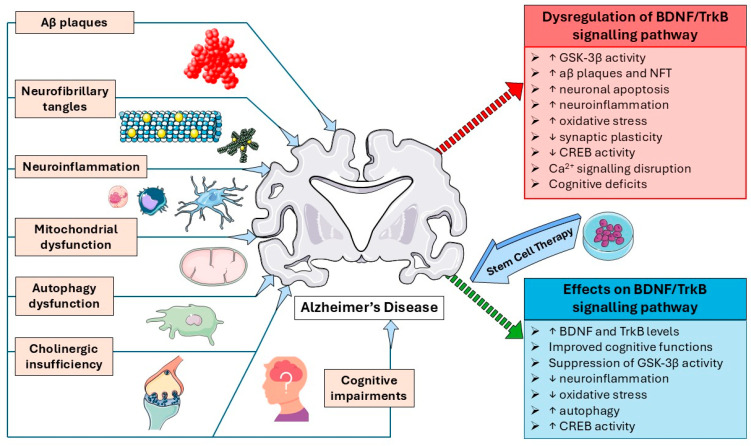
Pathophysiology of AD, dysregulation of BDNF/TrkB signalling pathway and the effects of stem cell therapy on BDNF/TrkB signalling pathway in AD. Image(s) adapted from Servier Medical Art (https://smart.servier.com/), licensed under CC BY 4.0 (https://creativecommons.org/licenses/by/4.0/).

From the evidence presented, stem cell therapy has shown to exert beneficial modulation effects on the BDNF/TrkB signalling pathway and its downstream cascades in mitigating AD by mainly upregulating BDNF and TrkB levels, suppressing GSK-3β activity, increasing autophagy and CREB activity, reducing neuroinflammation and oxidative stress, and improving cognitive functions as summarized in Table 1 and Figure 2.

## 5. Challenges and Limitations

A major challenge in establishing a therapeutic approach for AD is the disease modelling in the preclinical study phase. The discrepancy in translating and reflecting the preclinical findings into clinical trials is due to the complexity of the disease and the inability of replicating the human’s brain environment and its multifaceted relationship with other factors such as ageing, genetic and environmental factors in the AD models [125]. Developing a good and sustainable model of NDs is also challenging due to the complexity of the brain itself. On top of that, the diagnosis of AD is mostly confirmed after manifestation of multiple cognitive deficits that interfere with daily functioning of the patients for late-onset AD. By then, the molecular pathology of AD has been progressively developed over the years, complicating disease management. This highlights the importance in addressing the root causes of the disease by targeting molecular pathways or mechanisms.

In the case of stem cell therapy for AD, one of the major challenges is the delivery of stem cells into the brain itself due to the presence of BBB that selectively controls and limits the passage of molecules in and out of the brain [126]. Systemic delivery such as IV is less effective compared to direct delivery such as ICV, and most of the studies discussed above used ICV for the route of stem cell administration. However, it is still important to note that systemic delivery of intended drugs or therapeutic components is more advantageous for translation into clinical applications. Long-term survival of engrafted cells is also a key point to take into account to ensure the stability and efficacy of the treatment while evading the host’s immune response [127]. Decline graft optimization of engrafted cells in hosts could potentially be a serious issue as it may cause unpredictable interactions that might be harmful to the host. Potential tumorigenesis initiated by engrafted cells also possesses a major concern in cell-based therapy [128].

Precise targeting and homing of engrafted cells to the target area is also one of the challenges identified due to the progressive and widespread nature of AD that affects multiple brain regions, primarily the hippocampus and cerebral cortex, making it even harder to localize the engrafted cells into the target area. Apart from that, cell-based therapy has always been linked to ethical concerns, and optimization of donor cells as certain types of stem cells such as NSCs are limited [129]. In translating the use of stem cells in clinical settings, it is also important to have a standardized system and strict control in terms of cell production by following the current good manufacturing practice (cGMP) to ensure high quality and safe products for human use [130].

## 6. Future Directions

Detailed explorations focusing on the BDNF/TrkB pathway and its downstream cascades specifically is a good approach in elucidating the underlying mechanisms involved in AD progression and to design a holistic approach targeting this pathway. The use of BDNF mimetics and TrkB agonists such as 7,8-dihydroxyflavone (7,8-DHF) and ENT-A011 are also being explored in studies targeting the BDNF/TrkB signalling pathway as a therapeutic approach for AD [130,131]. In developing a robust model for NDs, there are emerging studies of using iPSC in developing brain organoids technology for ND modelling as it can provide closer mimicry to human brain architecture and functions [132].

In improving the effects of stem cell-based therapy for AD models, studies have proven that pre-treatment, modification or overexpression of BDNF into the engrafted stem cells pre-transplantation exhibit enhanced positive outcomes for cognitive functions and neuronal effects compared to traditional stem cell transplantations [112,113,133,134,135]. Integration of other beneficial molecules, substrates or functional genes into the stem cells or combining other treatments with stem cell therapy to create synergistic effects on the BDNF/TrkB pathway may enhance therapeutic outcomes in ameliorating AD. Nanoparticles, natural products such as curcumin, microRNAs, Nrf2 activator, asparaginyl endopeptidase and many more are examples of integrative treatments for AD [136,137,138,139,140,141].

The advancement of stem cell therapy by using extracellular vesicles such as exosomes in enhancing the targeted delivery for therapeutic interventions is also anticipated, as they possess the potential to augment cell proliferation, differentiation, migration and tissue regeneration, in addition to acting as an effective vehicle for transporting bioactive molecules [142]. These prospective developments of stem cell therapy are anticipated in developing a novel and effective therapeutic approach for AD. On top of that, intranasal delivery is also being explored as a route of administration to improve the distribution of the engrafted stem cells for treatment of AD [143,144,145,146,147,148].

## 7. Conclusions

The BDNF/TrkB pathway is a key regulator of other important downstream cascades that are crucial in neuronal survival, synaptic plasticity and learning and memory acquisition. The overview and dysregulation of the BNDF/TrkB signalling pathway and its downstream cascades, particularly the PI3K/Akt, MAPK/ERK and PLC-γ pathways, in AD are discussed in this review. Pieces of evidence from the studies of stem cell therapy have been shown to achieve therapeutic effects through upregulation of the BDNF/TrkB signalling pathway and enhancement of its downstream cascades, which lead to neuroprotective mechanisms and improved cognitive functions that contribute to mitigation of AD.

## Figures and Tables

**Table 1:** Summary of stem cell therapy effects on BDNF/TrkB signalling pathway and brief protocols of studies included.

Model	Stem Cell Type	Dose and Route of Administration	Key Findings (BDNF/TrkB and Downstream Cascades)	Ref.
In vivo C57BL/6 transgenic mice	NSCs isolated from mice (untreated + treated with BDNF)	1 × 10^6^ cells/μL Stereotactic transplantation	NSC+BDNF group significantly improved memory and learning ability; BDNF pretreatment improved NSC transplantation effects	[112]
In vivo Tg2576 transgenic mice	NSCs isolated from mice (untreated + treated with BDNF and knockdown BDNF)	5 × 10^4^ cells/μL Stereotactic transplantation	NSC and NSC+BDNF groups improved cognitive deficits with better performance from NSC+BDNF group; BDNF pretreatment improved NSC transplantation effects; BDNF knockdown blocked cognitive improvement	[113]
In vivo Tg2576 transgenic mice	Human UC-MSCs	2 × 10^6^ cells Intravenous injection	hUC-MSCs improved cognitive function and increased BDNF level significantly	[114]
In vivo 5xFAD transgenic mice	Induced neural progenitor/stem cells (iNPCs)	5 × 10^4^ cells/μL Stereotactic transplantation	iNPCs improved cognitive function and increased BDNF level in hippocampus	[115]
In vivo C57BL/6 transgenic mice	BMSC-exos isolated from mouse BMSCs	0.5 μg BMSC exos (dissolved in 2 μL ACSF) for ICV 25 μg BMSC exos (dissolved in 100 μL PBS) for IV	BMSC-exos via ICV group improved AD-like behaviours and significantly increased BDNF expression compared to BMSC-exos via IV group	[117]
In vivo APP/PS1 double transgenic mice	NSCs isolated from mice	5 × 10^5^ to 1 × 10^6^ in 5 μL Stereotactic transplantation	NSCs significantly restored spatial learning and memory deficits; NSCs increased the levels of BDNF and TrkB proteins and mRNA	[118]
In vivo 3xTg-AD transgenic mice	NSCs isolated from mice	1 × 10^5^ cells/μL Stereotactic transplantation	NSCs improved AD-related cognitive dysfunction; NSCs increased BDNF level significantly, without altering aβ and tau levels; BDNF knockdown within NSCs abolished cognitive recovery	[119]
In vivo NSE/APPsw transgenic mice	Human NSCs (hNSCs)	1 × 10^5^ cells/μL Stereotactic transplantation	hNSCs improved spatial memory; hNSC reduced tau phosphorylation via Trk-induced Akt/GSK3β signalling; hNSCs expressed BDNF that induce Trk-dependent Akt activation; hNSC induced significantly higher phosphorylation levels of TrkA/B and Akt and markedly elevated the level of GSK3β phosphorylation	[120]
In vivo Aβ25-35-induced during pregnancy Wistar rat dam	BMSCs isolated from rat’s bone marrow	1 × 10^6^ cells Intravenous injection	BMSCs significantly increased serum BDNF and BDNF mRNA and decreased serum GSK-3β levels	[121]
In vivo APP/PS1 double transgenic mice	BMSCs (isolated from mice’s bone marrow) + adenovirus carrying GFP-CX3CL1-Wnt3a (CX3CL1-Wnt3a-MSC)	5 × 10^4^ cells/μL Stereotactic transplantation	CX3CL1-Wnt3a-MSC significantly alleviated cognitive impairments, increased p-Akt and PI3K levels and elevated phosphorylation of GSK-3β at Ser9; CX3CL1-Wnt3a-MSC inhibited GSK-3β via PI3K/Akt pathway	[123]
In vivo 3xTg-AD transgenic mice	Human DPSCs (hDPSCs)	1 × 10^5^ in 5 μL PBS ICV injection	hDPSCs promoted the upregulation of p-AKT (ser473) and p-GSK-3β (ser9); hDPSCs ameliorated LPS-induced oxidative stress and apoptosis in BV2 cells by activating Nrf2 via the AKT/GSK-3β pathway	[124]
In vivo aluminum-induced Wistar rat	BMSCs isolated from rat’s bone marrow	1 × 10^6^ cells Intravenous injection	BMSCs ameliorated the downregulation of p-PI3K level; BMSCs inhibited GSK-3β via increasing the expression of the p-GSK-3β	[125]
In vivo aluminum-induced Albino rat	BMSC-exos isolated from rat’s BMSCs	0.5 mL of BMSC-exos (100 μg protein/mL) Intraperitoneal injection	BMSC-exos improved memory function; BMSC-exos effectively reduced the elevated levels of p-Akt/Akt and p- GSK-3β; MSC-exos together with autophagy inhibitors significantly reduced cerebral p-Akt/Akt and p- GSK-3β levels; BMSC-exos modulated AKT/mTOR signalling in the AD rat brain by decreasing mTOR expression and increasing AMPK expression	[126]
In vivo aluminum-induced Sprague-dawley rat	BMSCs isolated from rat’s bone marrow	3 × 10^6^ cells Intravenous injection	BMSC transplantation significantly enhanced p-Akt protein expression; BMSC transplantation significantly increased p-ERK1/2 protein expression	[127]
In vivo and in vitro aβ-induced AD mice and hippocampal cell culture	BMSCs isolated from mice’s bone marrow and hippocampal neurons from E18 C57BL/6 mice	1 × 10^5^ cells Stereotactic transplantation (in vivo) Co-incubation with BMSCs for 24 h (in vitro)	In vivo: BMSCs treatment improved learning and memory In vitro: BMSCs significantly increased CREB and ERK phosphorylation; BM-MSCs mediated protection against aβ-induced apoptosis via activation of the MAPK/ERK pathway	[128]
In vivo aβ-induced Swiss albino mice	Lin^−^ stem cells were isolated from mono-nucleated cell population of human UCB samples	5 × 10^4^ or 1 × 10^5^ cells Stereotactic transplantation	hUCB Lin^−^ stem cells could potentially reverse aβ-induced cognitive impairment through a neuroprotective mechanism mediated by CREB and BDNF	[129]
In vitro aβ_1−42_-treated neural cells	BMSCs isolated from rat’s bone marrow	Co-culture with BMSCs with ratio of cells 1:1	BMSCs significantly decreased protein expression levels of p-AMPK, mTOR, p-mTOR and GSK-3β; BMSCs significantly increased levels of p-GSK-3β, Wnt3, and β-catenin; BMSCs’ effects on aβ-treated neural cells showed positive effects through modulation of mTOR, AMPK, GSK-3β and Wnt/β-catenin pathways	[122]

## Data Availability

No new data were created or analyzed in this study.
